# Trends in Mortality From Poisonings, Firearms, and All Other Injuries by Intent in the US, 1999-2020

**DOI:** 10.1001/jamainternmed.2023.2509

**Published:** 2023-07-03

**Authors:** Wayne R. Lawrence, Neal D. Freedman, Jennifer K. McGee-Avila, Amy Berrington de González, Yingxi Chen, Marc A. Emerson, Gilbert C. Gee, Emily A. Haozous, Anika T. Haque, Maki Inoue-Choi, Sarah S. Jackson, Brittany Lord, Anna M. Nápoles, Eliseo J. Pérez-Stable, Jacqueline B. Vo, Faustine Williams, Meredith S. Shiels

**Affiliations:** 1 Division of Cancer Epidemiology and Genetics, National Cancer Institute, National Institutes of Health, Rockville, Maryland; 2 Division of Genetics and Epidemiology, The Institute of Cancer Research, London, United Kingdom; 3 Department of Epidemiology, University of North Carolina at Chapel Hill, Chapel Hill; 4 Department of Community Health Sciences, Fielding School of Public Health, University of California Los Angeles, Los Angeles; 5 Pacific Institute for Research and Evaluation, Albuquerque, New Mexico; 6 Division of Intramural Research, National Institute on Minority Health and Health Disparities, National Institutes of Health, Bethesda, Maryland; 7 Office of the Director, National Institute on Minority Health and Health Disparities, Bethesda, Maryland

## Abstract

**Importance:**

Although deaths due to external causes are a leading cause of mortality in the US, trends over time by intent and demographic characteristics remain poorly understood.

**Objective:**

To examine national trends in mortality rates due to external causes from 1999 to 2020 by intent (homicide, suicide, unintentional, and undetermined) and demographic characteristics. External causes were defined as poisonings (eg, drug overdose), firearms, and all other injuries, including motor vehicle injuries and falls. Given the repercussions of the COVID-19 pandemic, US death rates for 2019 and 2020 were also compared.

**Design, Setting, and Participants:**

Serial cross-sectional study using national death certificate data obtained from the National Center for Health Statistics and including all external causes of 3 813 894 deaths among individuals aged 20 years or older from January 1, 1999, to December 31, 2020. Data analysis was conducted from January 20, 2022, to February 5, 2023.

**Exposures:**

Age, sex, and race and ethnicity.

**Main Outcomes and Measures:**

Trends in age-standardized mortality rates and average annual percentage change (AAPC) in rates calculated by intent (suicide, homicide, unintentional, and undetermined), age, sex, and race and ethnicity for each external cause.

**Results:**

Between 1999 and 2020, there were 3 813 894 deaths due to external causes in the US. From 1999 to 2020, poisoning death rates increased annually (AAPC, 7.0%; 95% CI, 5.4%-8.7%). From 2014 to 2020, poisoning death rates increased the most among men (APC, 10.8%; 95% CI, 7.7%-14.0%). During the study period, poisoning death rates increased in all the racial and ethnic groups examined; the most rapid increase was among American Indian and Alaska Native individuals (AAPC, 9.2%; 95% CI, 7.4%-10.9%). During the study period, death rates for unintentional poisoning had the most rapid rate of increase (AAPC, 8.1%; 95% CI, 7.4%-8.9%). From 1999 to 2020, firearm death rates increased (AAPC, 1.1%; 95% CI, 0.7%-1.5%). From 2013 to 2020, firearm mortality increased by an average of 4.7% annually (95% CI, 2.9%-6.5%) among individuals aged 20 to 39 years. From 2014 to 2020, mortality from firearm homicides increased by an average of 6.9% annually (95% CI, 3.5%-10.4%). From 2019 to 2020, mortality rates from external causes accelerated further, largely from increases in unintentional poisoning, and homicide due to firearms and all other injuries.

**Conclusions and Relevance:**

Results of this cross-sectional study suggest that from 1999 to 2020, death rates due to poisonings, firearms, and all other injuries increased substantially in the US. The rapid increase in deaths due to unintentional poisonings and firearm homicides is a national emergency that requires urgent public health interventions at the local and national levels.

## Key Points

**Question:**

How did US adult mortality rates due to external causes change between 1999 and 2020 by age, sex, race and ethnicity, and intent, and from 2019 to 2020 (after the onset of COVID-19)?

**Findings:**

In this cross-sectional study of 3 813 894 deaths due to external causes, deaths from poisonings, firearms, and all other injuries increased between 1999 and 2020. From 2019 to 2020, death rates from each cause further accelerated, with the largest increases for deaths from unintentional poisoning and firearm deaths due to homicide.

**Meaning:**

The findings suggest that deaths due to external causes are increasing in the US.

## Teaser

This cross-sectional study reports trends in mortality from poisonings, firearms, and all other injuries by intent in US adults from 1999 to 2020.

## Introduction

During the past decade, life expectancy at birth in the US has changed little in contrast to that in other high-income countries, where life expectancy has increased. In 2020, however, life expectancy at birth in the US decreased considerably because of the COVID-19 pandemic.^1,2^ External causes of death, including both intentional and unintentional injury and poisoning (eg, drug overdose) mortality, have contributed substantially to recent decreases in life expectancy.^3,4,5,6^ Studies have suggested the COVID-19 pandemic was associated with a further increase in deaths from external causes as a result of socioeconomic hardship, psychosocial stress, and reduced access to health care.^7,8,9^ Nonetheless, changes in demographic trends over time and differences in rates of external causes of death by intent (ie, suicide, homicide, and unintentional) and by sex, age, and race and ethnicity are not fully understood.^7,8^

In the US, the risk of external causes of deaths varies across sociodemographic characteristics.^3,10^ Although deaths due to firearms and poisoning are increasing nationally, the trends for suicide, homicide, and unintentional injury may differ by age, sex, and race and ethnicity.^7,8,9,11,12,13^ During the past 2 decades, deaths due to drug overdose have emerged as a major public health concern and had been an important contributor to plateauing US life expectancy before the COVID-19 pandemic.^3,5^ Drug overdose deaths, however, have disproportionally affected White individuals, although recent evidence suggests that rates are increasing among other racial and ethnic groups, such as Black and Hispanic or Latino individuals.^3,5,9^

In this study, we describe trends in mortality among adults in the US from 1999 to 2020 for poisonings, firearms, and all other injuries by intent, sex, age, and race and ethnicity. We further evaluated trends in poisoning deaths due to drug overdose and all other injury deaths, including those due to motor vehicle injuries and falls.

## Methods

### Data Source and Population Characteristics

For this cross-sectional study, we obtained information about demographic characteristics and underlying causes of death from death certificate data compiled by the National Center for Health Statistics from January 1, 1999, to December 31, 2020. Because our analyses focused on adults, we restricted the study to individuals aged 20 years or older.^10,14,15,16^ We categorized external causes of deaths into 3 types: poisonings, firearms, and all other injuries (ie, causes not involving firearm or poisoning). We then categorized each type of external cause of death by intent (unintentional, homicide, suicide, and undetermined) according to *International Statistical Classification of Diseases and Related Health Problems, 10th Revision* codes (eTable 1 in Supplement 1).^17^ Because the leading cause of death during the 1999-2020 period for poisoning was drug overdose (912 670 of 1 000 340 [91.2%]) and for all other injuries was falls (589 641 of 2 143 494 [27.5%]) and motor vehicle–related deaths (640 425 of 2 143 494 [30.0%]), we further examined deaths due to these specific causes (eTable 2 in Supplement 1). We stratified population estimates of deaths in each category of external causes of death by year, age, sex, and race and ethnicity.^16^ Race and ethnicity were categorized using death certificate data as non-Hispanic American Indian and Alaska Native, non-Hispanic Asian and Pacific Islander, non-Hispanic Black (Black), non-Hispanic White (White), and Hispanic or Latino. Death rates among American Indian and Alaska Native individuals were restricted to Indian Health Service Purchased/Referred Care Delivery Areas to increase the sensitivity of ascertainment of American Indian and Alaska Native race on death certificates.^18^ Because all data were publicly available, institutional review board approval was not required. This study followed the Strengthening the Reporting of Observational Studies in Epidemiology (STROBE) reporting guideline.^19^

### Statistical Analysis

We calculated age-standardized mortality rates by age group (20-39, 40-64, and ≥65 years), race and ethnicity, sex, type of external cause of death, and intent, using SEER*Stat, version 8.4.0 (National Cancer Institute). All rates were age standardized to the US population in 2000 and presented as per 100 000 population.

We used the Joinpoint Regression Program to calculate the average annual percentage change in mortality rates, representing the summary measure from 1999 to 2020, stratified by age, sex, race and ethnicity, type of external cause of death, and intent. Using Joinpoint regression, we also calculated annual percentage changes (APCs); in this analysis, we identified calendar years with significant changes in trajectories and calculated the slope in each segment. We used the parametric method to calculate 95% CI for trend. *P* values were calculated with the permutation distribution of the test statistic (*P* < .05 was considered statistically significant, with a 2-sided test). Given the potentially large change in death rates due to the COVID-19 pandemic in 2020, which may not be captured in the average annual percentage changes and APCs, rate ratios (RRs) for each external cause of death overall and by intent were calculated for comparison between 2019 and 2020. Data analysis was conducted from January 20, 2022, to February 5, 2023.

## Results

Between 1999 and 2020, there were 3 813 894 deaths due to external causes in the US. The number of deaths due to external causes increased from 65.6 (per 100 000 population) in 1999 to 103.5 in 2020. From 1999 to 2020, the largest absolute increase in mortality rates were for poisoning (31.18 per 100 000 population), followed by firearm injuries (4.18 per 100 000 population) and all other injuries (2.57 per 100 000 population) (eTable 3 in Supplement 1).

**Figure 1.**
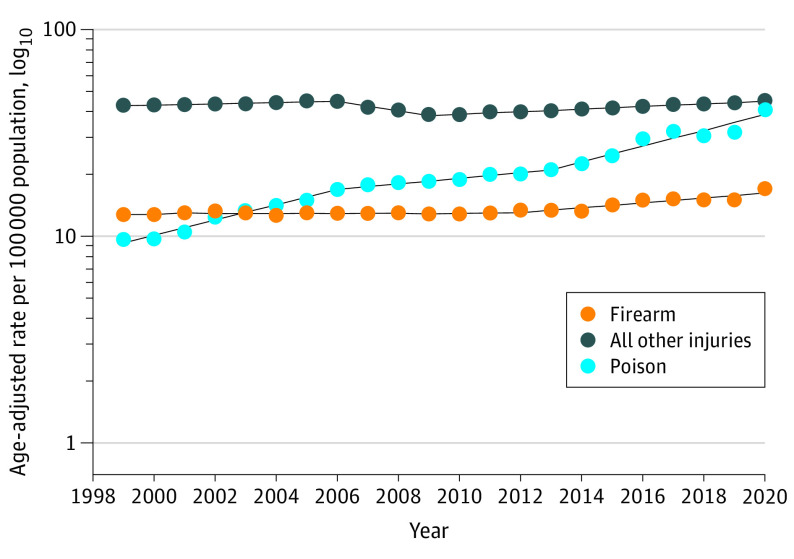
Trends in Age-Standardized Mortality Rates Due to External Causes in the US, 1999-2020 The dashed lines indicate modeled age-adjusted rates, and the circles indicate observed age-adjusted rates.

**Figure 2.**
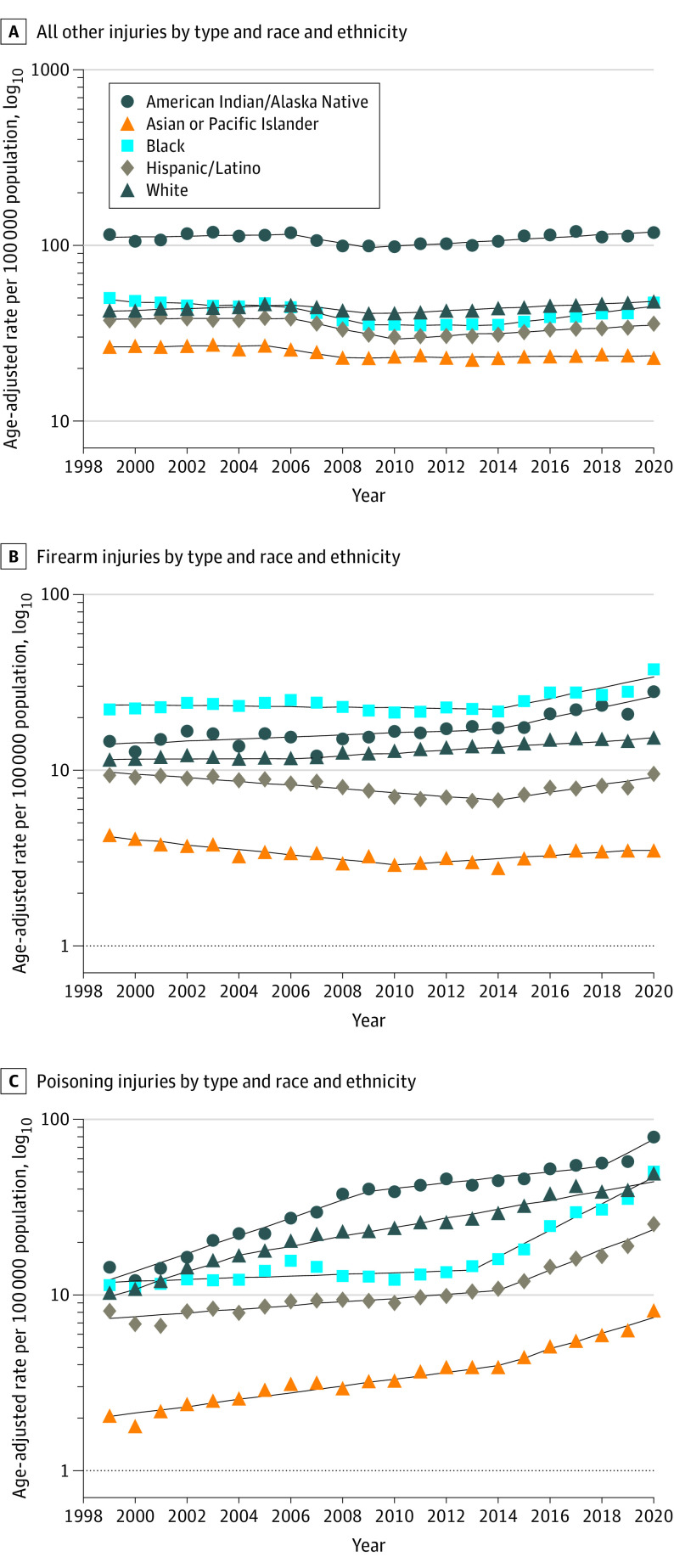
Trends in Age-Standardized Mortality Rates Due to External Causes by Type and Race and Ethnicity in the US, 1999-2020 The dashed lines indicate modeled age-adjusted rates, and the circles, triangles, squares, and diamonds indicate observed age-adjusted rates.

Poisoning death rates increased 7.0% annually (95% CI, 5.4%-8.7%), with the most rapid increase observed from 2013 to 2020 (APC, 9.1%; 95% CI, 6.9%-11.4%) (Figure 1). Drug overdose deaths, which accounted for most poisoning deaths, exhibited a similar trend from 2013 to 2020 (APC, 10.0%; 95% CI, 7.6%-12.4%) (eFigure 1 in Supplement 1). From 2014 to 2020, poisoning death rates increased rapidly among men (APC, 10.8%; 95% CI, 7.7%-14.0%) (eFigure 2 in Supplement 1). For all age groups, there was an increasing trend in poisoning death rates (eFigure 3 in Supplement 1). Additionally, poisoning deaths increased across all racial and ethnic groups, with the largest annual increase among American Indian and Alaska Native persons (average annual percentage change, 9.2%; 95% CI, 7.4%-10.9%) (Figure 2; eTable 4 in Supplement 1). Between 2014 and 2020, there was a sharp increase in poisoning deaths among Asian and Pacific Islander persons (APC, 11.1%; 95% CI, 8.7%-13.6%). When examined by intent, although unintentional poisoning deaths increased by 8.1% per year (95% CI, 7.4%-8.9%) from 1999 to 2020, suicide poisoning mortality rates increased from 1999 to 2008 (APC, 2.0%; 95% CI, 1.5%-2.5%) and decreased from 2008 to 2016 and more rapidly between 2016 and 2020 (APC, −5.4%; 95% CI, −7.0% to −3.8%) (eTable 5 in Supplement 1).

**Figure 3.**
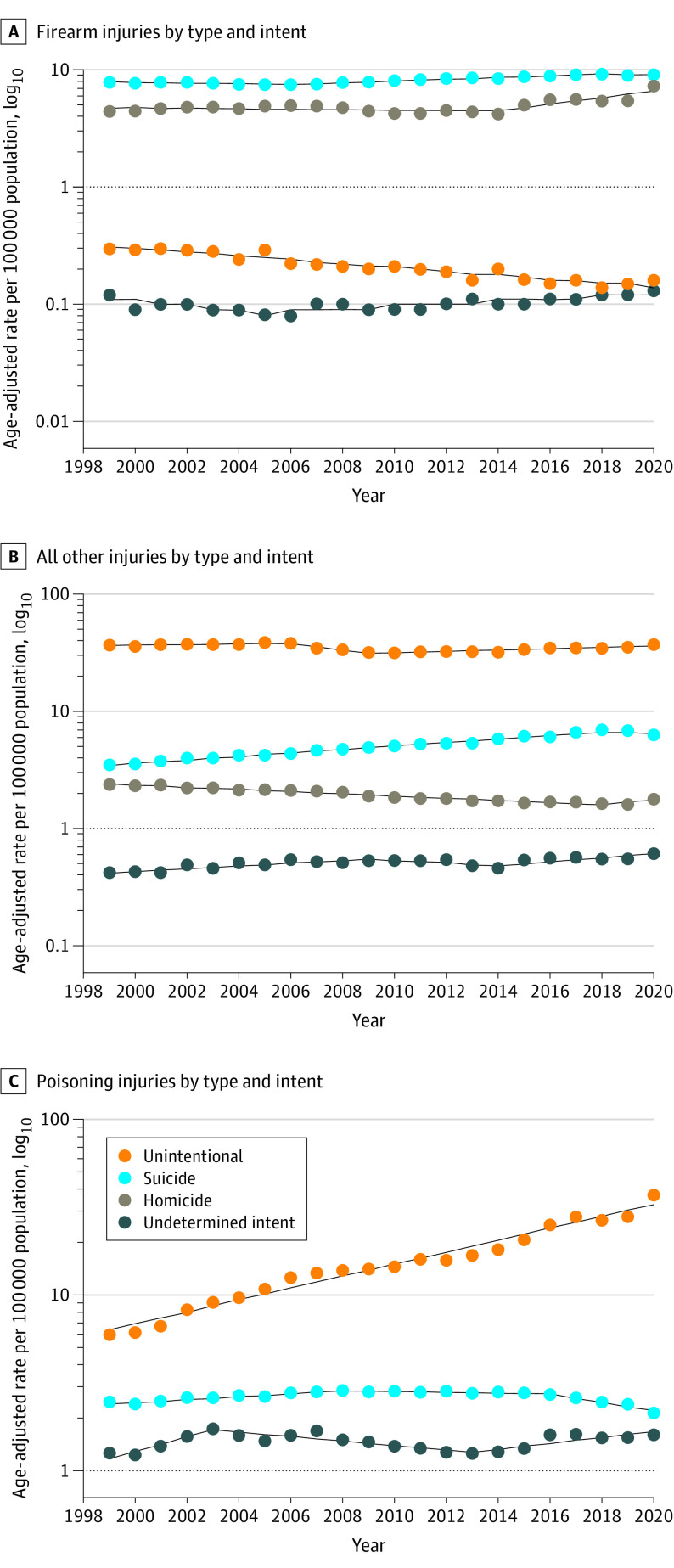
Trends in Age-Standardized Mortality Rates Due to External Causes by Type and Intent in the US, 1999-2020 The dashed lines indicate modeled age-adjusted rates, and the circles indicate observed age-adjusted rates.

From 1999 to 2020, firearm death rates increased 1.1% annually (95% CI, 0.7%-1.5%) (Figure 1). There was an increase in firearm death rates of 3.0% per year (95% CI, 2.5%-10.6%) among men from 2013 to 2020 and 2.5% per year (95% CI, 2.1%-2.9%) among women from 2008 to 2020 (eTable 6 in Supplement 1). Additionally, firearm deaths increased sharply annually from 2013 to 2020 among individuals aged 20 to 39 years (APC, 4.7%; 95% CI, 2.9%-6.5%) (eTable 7 in Supplement 1). Black persons had the highest rates of firearm deaths throughout the study period (Figure 2); for example, rates among Black individuals were more than 5 times higher than those of Asian and Pacific Islander persons (24.5 vs 3.3 per 100 000 population, respectively) (Table). However, American Indian and Alaska Native individuals experienced the most rapid increase in firearm death rates (average annual percentage change, 3.0%; 95% CI, 1.6%-4.4%) (eTable 4 in Supplement 1). From 2014 to 2020, mortality from firearm homicides increased by an average of 6.9% annually (95% CI, 3.5%-10.4%). Firearm suicide rates decreased from 1999 to 2006 (APC, −0.7%; 95% CI, −1.2% to −0.2%), increased from 2006 to 2018 (APC, 1.7%; 95% CI, 1.5%-1.9%), and then decreased from 2018 to 2020 (APC, −0.9%; 95% CI, −3.9% to 2.3%) (Figure 3; eTable 5 in Supplement 1). Moreover, unintentional firearm death rates decreased by 3.7% per year (95% CI, −4.3% to −3.2%) from 1999 to 2020.

**Table.** Age-Standardized Mortality Rates by Type of External Cause of Death in the US, 1999-2020

By type and intent	Age-standardized death rate per 100 000 population (95% CI)^a^					
	Non-Hispanic individuals					Hispanic or Latino individuals
	Total population	American Indian or Alaska Native^b^	Asian or Pacific Islander	Black	White	
Poisoning	20.7 (20.7-20.8)	38.3 (37.5-39.1)	3.9 (3.9-4.0)	19.0 (19.0-19.1)	24.6 (24.5-24.6)	11.9 (11.8-12.0)
Unintentional	16.6 (16.5-16.6)	33.5 (32.7-34.2)	2.7 (2.6-2.8)	16.4 (16.3-16.5)	19.3 (19.2-19.3)	10.5 (10.4-10.6)
Suicide	2.7 (2.6-2.7)	3.1 (2.9-3.4)	1.0 (1.0-1.0)	0.9 (0.9-0.9)	3.4 (3.4-3.5)	0.9 (0.9-1.0)
Homicide	0.03 (0.03-0.03)	0.10 (0.02-0.10)	0.01 (0.01-0.02)	0.04 (0.04-0.10)	0.03 (0.03-0.04)	0.02 (0.01-0.02)
Undetermined	1.5 (1.5-1.5)	1.7 (1.5-1.9)	0.2 (0.2-0.2)	17.8 (17.7-17.9)	1.8 (1.8-1.8)	0.4 (0.4-0.5)
Firearm	13.6 (13.6-13.7)	17.5 (17.0-18.1)	3.3 (3.3-3.4)	24.5 (24.4-24.7)	12.8 (12.8-12.8)	8.0 (8.0-8.1)
Unintentional	0.2 (0.2-0.2)	0.4 (0.4-0.5)	0.04 (0.03-0.10)	0.3 (0.3-0.3)	0.2 (0.2-0.2)	0.1 (0.1-0.1)
Suicide	8.3 (8.2-8.3)	10.3 (9.9-10.7)	1.8 (1.7-1.8)	3.9 (3.9-4.0)	10.4 (10.3-10.4)	3.1 (3.0-3.1)
Homicide	4.9 (4.9-4.9)	5.8 (5.5-6.1)	1.4 (1.4-1.5)	19.9 (19.8-20.0)	2.0 (1.9-2.0)	4.6 (4.5-4.6)
Undetermined	0.1 (0.1-0.1)	0.3 (0.3-0.4)	0.03 (0.02-0.03)	0.1 (0.1-0.1)	0.1 (0.1-0.1)	0.1 (0.1-0.1)
All other injuries	42.5 (42.5-42.6)	109.2 (107.7-110.6)	24.1 (23.9-24.3)	41.0 (40.8-41.1)	44.2 (44.1-44.3)	34.1 (34.0-34.3)
Unintentional	35.1 (35.0-35.1)	85.7 (84.4-87.0)	17.8 (17.6-18.0)	32.7 (32.6-32.9)	36.7 (36.6-36.8)	27.7 (27.5-27.8)
Suicide	5.1 (5.1-5.1)	12.4 (12.0-12.9)	5.0 (4.9-5.1)	2.7 (2.7-2.8)	5.8 (5.8-5.8)	3.9 (3.8-3.9)
Homicide	1.9 (1.9-2.0)	8.5 (8.1-8.9)	1.0 (0.9-1.0)	5.1 (5.0-5.1)	1.3 (1.3-1.3)	2.2 (2.2-2.3)
Undetermined	0.5 (0.5-0.5)	2.8 (2.5-3.0)	0.4 (0.3-0.4)	0.8 (0.7-0.8)	0.5 (0.5-0.5)	0.5 (0.4-0.5)
Motor vehicle related^c^	13.0 (12.9-13.0)	40.1 (39.3-40.9)	5.8 (5.7-5.9)	14.7 (14.6-14.8)	13.2 (13.1-13.2)	12.0 (11.9-12.1)
Falls^c^	11.3 (11.2-11.3)	16.3 (15.6-16.9)	7.6 (7.5-7.7)	5.9 (5.8-6.0)	12.1 (12.1-12.2)	8.2 (8.1-8.2)

^a^ Rates are per 100 000 population and age adjusted to the 2000 US standardized population.

^b^ Data for the non-Hispanic American Indian or Alaska Native population are restricted to Indian Health Service Purchased/Referred Care Delivery Area counties.

^c^ Includes deaths reported in other “all other injuries” categories.

Death rates due to all other injuries increased from 1999 to 2006 (APC, 0.7%; 95% CI, 0.2%-1.2%) and then decreased from 2006 to 2009 (APC, −5.2%; 95% CI, −8.9% to −1.3%) before increasing from 2009 to 2020 (APC, 1.5%; 95% CI, 1.2%-1.7%) (eTable 5 in Supplement 1). Motor vehicle–related deaths accounted for a large proportion of these deaths, and the annual death rate increased by an average of 1.1% (95% CI, 0.3%-1.8%) from 2010 to 2020 (eTable 8 in Supplement 1). From 2010 to 2020, the increase in motor vehicle–related death was greatest among men (APC, 1.2%; 95% CI, 0.4%-2.0%) and individuals aged 40 to 64 years (APC, 1.6%; 95% CI, 0.9%-2.4%) (eTable 8 in Supplement 1). When examined by race and ethnicity, the most rapid increase was among Black individuals from 2011 to 2020 (APC, 5.0%; 95% CI, 3.5%-6.4%). Deaths due to falls increased by 3.6% on average annually from 1999 to 2020 (eTable 9 in Supplement 1) and was most rapid among individuals aged 65 years or older, increasing by 4.2% per year (95% CI, 3.9%-4.4%) from 1999 to 2020. Moreover, the largest increase was among White individuals from 2007 to 2020 (APC, 3.0%; 95% CI, 2.8%-3.3%).

**Figure 4.**
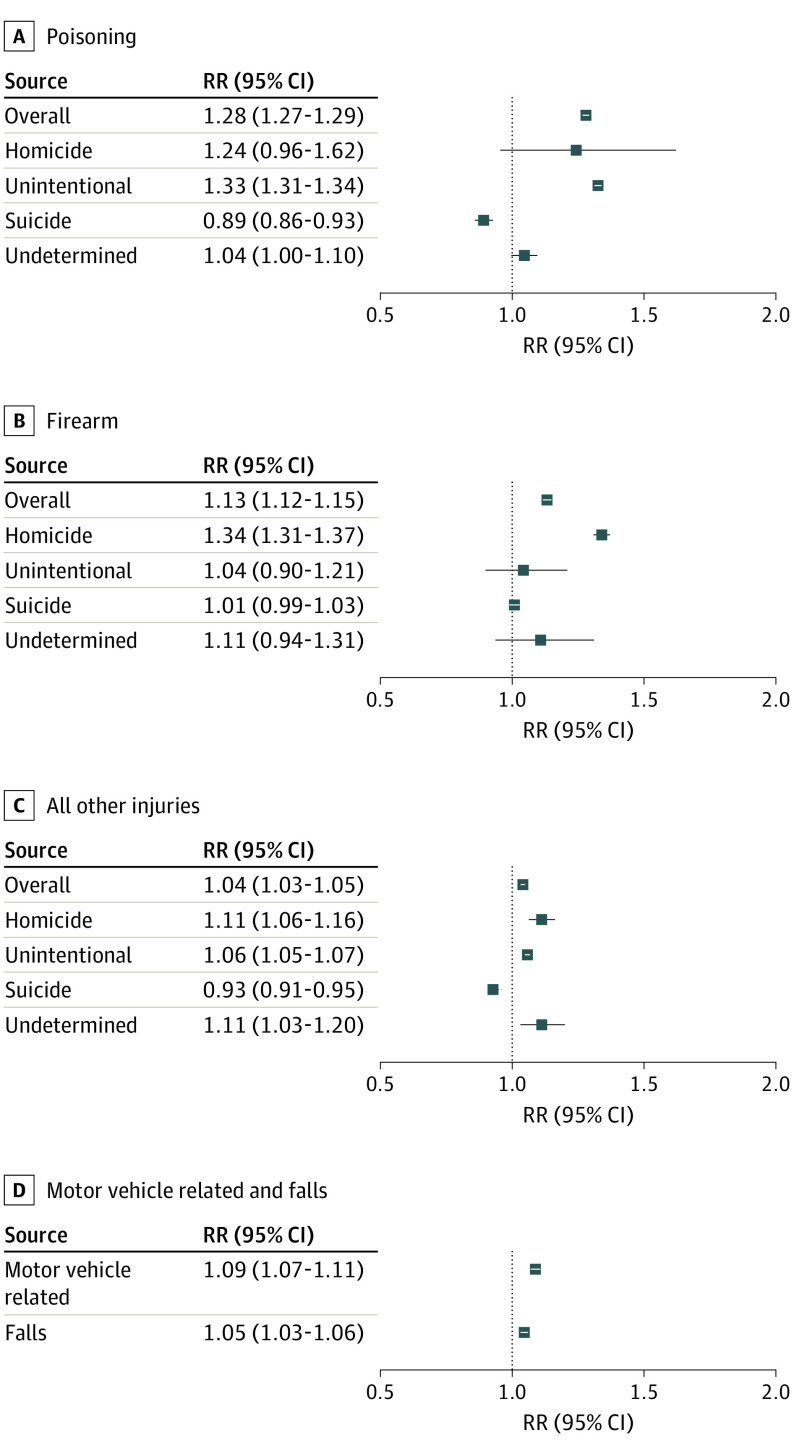
Age-Adjusted Death Rate Ratio Owing to External Causes by Type Between 2019 and 2020 in the US The referent group is 2019. RR indicates rate ratio.

From 2019 to 2020, death rates from poisonings, firearms, and all other injuries increased, with the largest increases observed for poisoning deaths (RR, 1.28; 95% CI, 1.27-1.29; absolute change, 8.92 per 100 000 population) (Figure 4). These increases exceeded the average increases in the preceding years. For instance, the 28% increase from 2019 to 2020 in the poisoning death rate was 3 times greater than the average annual increase from 2013 to 2020 (eTable 5 in Supplement 1). By intent, the largest relative increases in death rates were firearm-involved homicides (RR, 1.34; 95% CI, 1.31-1.37; absolute change, 1.84) and unintentional poisonings (RR, 1.33; 95% CI, 1.31-1.34; absolute change, 9.10). Additionally, there was an increase in motor vehicle–related deaths (RR, 1.09; 95% CI, 1.07-1.11). In contrast, there was a reduced suicide rate from poisonings (RR, 0.89; 95% CI, 0.86-0.93).

## Discussion

In a national analysis of data from death certificates, we identified increasing mortality from poisonings, firearms, and all other injuries in the US from 1999 to 2020, with patterns varying by intent. Although the rate of change differed, these trends were generally observed across all racial and ethnic groups. Moreover, we observed large increases in mortality due to external causes from 2019 to 2020, likely reflecting indirect results of the COVID-19 pandemic. Our findings underscore the importance of targeted public health interventions at the local, state, and national levels to address these findings.

Between 1999 and 2020, the absolute increase in the poisoning death rate, primarily drug overdoses, was more than 7 times the increase in death rates due to firearms and all other injuries. Death rates due to poisoning more than tripled between 1999 to 2020, nearly overtaking all other injuries as the leading external cause. The initial increase in unintentional poisonings was originally attributed to the high per capita use of prescription opioids.^3,20^ However, opioid prescribing has largely decreased in recent years, and synthetic opioids and other substances are more frequently used.^3,20^ In 2013, deaths due to illicitly manufactured fentanyl and fentanyl analogues, which are potent synthetic opioids, started to increase rapidly and are at present the leading cause of opioid overdose death.^21,22^ Nationally, since 2008, poisoning death rates from suicide have decreased annually, which may reflect easier access to quicker lethal means, such as firearms.^23,24^ For instance, attempted suicide by firearm is largely irreversible; therefore, the majority of attempts are fatal compared with drug overdose because individuals have more time to reconsider their actions, summon help, or be discovered by someone before death.^23^ The increasing availability of naloxone may have also resulted in reduction in completing suicide attempts by drug overdose. Additionally, prior studies have reported that the rate of adult poisoning deaths is greater among men than women; our study observed that the rates further accelerated among men from 2014 to 2020.^3,25^

American Indian and Alaska Native persons consistently had the highest mortality rates due to poisoning, although there was a sharp increase from 2014 to 2020 among Black and Hispanic or Latino individuals. American Indian and Alaska Native communities experience significant barriers to accessing treatment for substance use disorders, including resources to prevent drug overdoses.^26^ The barriers to treatment are potentially exacerbated by the limited availability of mental health services because of underfunding of tribal clinics.^26,27,28^ The potential leading factors for greater poisoning deaths among American Indian and Alaska Native, Black, and Hispanic or Latino people also include historical trauma, systemic inequities, and structural racism, all of which are associated with greater exposure to psychosocial stressors and with living in communities that are socioeconomically disadvantaged.^26,27,29^ Additionally, we observed an increase in poisoning deaths among Asian and Pacific Islander persons from 2014 to 2020. Prior studies have indicated potential underreporting of substance use disorder and lower receipt of treatment among Asian and Pacific Islander people compared with most other racial groups.^29,30^

Firearm mortality rates increased throughout the study period. In recent years, homicide rates have increased substantially and are an important public health concern. The surge of firearm sales during the COVID-19 pandemic, especially to new gun owners, raises concerns about further increase in firearm deaths.^31^ In contrast, we observed an annual decrease in unintentional firearm deaths. State firearm laws and the promotion of safer gun storage may provide a partial explanation of our findings.^32,33^ Further research in this area is needed, particularly among adults.

Firearm death rates were consistently higher among Black individuals than other racial and ethnic groups, which may reflect the associations with neighborhood environments and structural racism.^34,35^ As a result of residential segregation, Black individuals disproportionally reside in neighborhoods that experience high crime rates, lower socioeconomic mobility, and greater psychosocial stressors; these neighborhoods may have higher concentrations of people who have incomes below the federal poverty level and are thus at greater risk of firearm violence.^34,35,36^ Additionally, we observed an increase in firearm death rates among American Indian and Alaska Native individuals. Recent studies have reported that American Indian and Alaska Native persons have higher rates of firearm deaths due to suicide and homicide than most other racial and ethnic groups; these rates are potentially associated with economic and social stressors, barriers to accessing mental health services, and interracial violence.^12,13,37,38,39^

Since 2009, mortality due to all other injuries not involving poisoning and firearms has increased. Motor vehicle–related deaths are one of the leading causes of injury deaths; from 2010 to 2020, we observed an annual average increase in rates. An increase in distracted driving may be associated with the increase in motor vehicle–related deaths, such as cellphone use.^40^ Additionally, Black individuals had the highest increases in motor vehicle–related deaths. A potential explanation is underinvestment in pedestrian infrastructure in predominantly Black neighborhoods, where traffic fatality risk per mile traveled has been reported to be greater compared with predominantly White neighborhoods.^41^ We further observed that deaths due to falls were greater and increasing annually among older adults. During the study period, the population of adults older than 65 years increased. Older adults are at greater risk of falls because of functional decline and chronic health conditions.^42,43^

COVID-19 was the third highest cause of death in the US in 2020 (the first was heart disease and the second was cancer) and indirectly led to increases in external causes of death.^7,8,44^ Mortality rates from poisonings, firearms, and all other injuries increased from 2019 to 2020, perhaps reflecting psychosocial stress and economic hardship associated with the pandemic, including increases in drug use, risky behaviors, and exposure to violence.^45^ Moreover, outreach workers who intervene in violent disputes were often unable to work in person, thereby hindering prevention efforts.^46^ The increase in motor vehicle–related deaths, despite lower traffic volume than in prior years, may reflect an increase in speeding and other risky behaviors among drivers.^45^ Our findings of reduced suicide rates in 2020 compared with 2019 despite documented increases in mental health disorders are consistent with prior findings.^9,47^ A potential explanation is that the pandemic relief actions taken by governmental organizations (eg, federal stimulus payments, moratorium on housing eviction) may have buffered economic hardship, thereby temporarily reducing psychosocial stress.^9^

### Limitations

A major limitation of the study is that the cause of death may have been incorrectly classified (eg, unintentional death instead of suicide), especially for poisoning deaths. Racial biases in death reporting (eg, underreporting suicide) are also well documented and could have been exacerbated during the COVID-19 pandemic. The study focused only on adults aged 20 years and older; therefore, our findings may not be generalizable to younger people. Additionally, we were unable to include deaths from 2021 owing to changes in racial and ethnic classification in death certificate data. Moreover, the data did not allow for assessing Asian and Pacific Islander racial groups separately, which is important because of distinct mortality patterns between the groups and the increase in anti-Asian racism.^48^

## Conclusion

This cross-sectional study found that from 1999 to 2020, death rates due to poisonings, firearms, and all other injuries increased substantially in the US. The study findings underscore the urgency of addressing the structural determinants of deaths from firearms, drug overdose, and other injuries, especially among racial and ethnic minoritized populations and their communities. Strategic and effective multilevel public health interventions are needed to counteract the increasing national trends in mortality due to external causes. In particular, the rapid increase in deaths due to unintentional poisonings and firearm homicides is a national emergency that requires urgent attention at the local and national levels.

## Notes

Supplement 1. **eTable 1.** External Causes of Death ICD-Codes **eTable 2.** Leading Causes of Poisoning and All Other Injuries Deaths From 1999-2020 **eTable 3.** Absolute Differences in External Causes of Death by Sex, Age Group, Race and Ethnicity, and Intent, 1999-2019 **eTable 4.** Annual Percentage Changes in Death Rates Due to External Causes by Race and Ethnicity, United States, 1999 to 2020 **eTable 5.** Annual Percentage Changes in Death Rates Due to External Causes by Intent, United States, 1999 to 2020 **eTable 6.** Annual Percentage Changes in Death Rates Due to External Causes by Sex, United States, 1999 to 2020 **eTable 7.** Annual Percentage Changes in Death Rates Due to External Causes by Age Group, United States, 1999 to 2020 **eTable 8.** Annual Percentage Changes in Motor Vehicle-Related Death Rates by Sex, Age Group, and Race and Ethnicity, United States, 1999 to 2020 **eTable 9.** Annual Percentage Changes in Fall Death Rates by Sex, Age Group, and Race and Ethnicity, United States, 1999 to 2020 **eFigure 1.** Trends in Age-Standardized Mortality Rates Due to Drug Poisoning by Intent, Unites States, 1999-2020. **eFigure 2.** Trends in Age-Standardized Mortality Rates Because of External Causes by Sex and Intent, United States, 1999 to 2020 **eFigure 3.** Trends in Age-Standardized Mortality Rates Because of External Causes by Age Group, United States, 1999 to 2020

Supplement 2.
Data Sharing Statement
