# Supplementary material for: Trends in Mortality From Poisonings, Firearms, and All Other Injuries by Intent in the US, 1999-2020
Source: JAMA Intern Med. 2023 Jul 3;183(8):849–56. doi: 10.1001/jamainternmed.2023.2509 (PMC10318548; doi:10.1001/jamainternmed.2023.2509)
Supplement: Supplement 1. — eTable 1. External Causes of Death ICD-Codes eTable 2. Leading Causes of Poisoning and All Other Injuries Deaths From 1999-2020 eTable 3. Absolute Differences in External Causes of Death by Sex, Age Group, Race and Ethnicity, and Intent, 1999-2019 eTable 4. Annual Percentage Changes in Death Rates Due to External Causes by Race and Ethnicity, United States, 1999 to 2020 eTable 5. Annual Percentage Changes in Death Rates Due to External Causes by Intent, United States, 1999 to 2020 eTable 6. Annual Percentage Changes in Death Rates Due to External Causes by Sex, United States, 1999 to 2020 eTable 7. Annual Percentage Changes in Death Rates Due to External Causes by Age Group, United States, 1999 to 2020 eTable 8. Annual Percentage Changes in Motor Vehicle-Related Death Rates by Sex, Age Group, and Race and Ethnicity, United States, 1999 to 2020 eTable 9. Annual Percentage Changes in Fall Death Rates by Sex, Age Group, and Race and Ethnicity, United States, 1999 to 2020 eFigure 1. Trends in Age-Standardized Mortality Rates Due to Drug Poisoning by Intent, Unites States, 1999-2020. eFigure 2. Trends in Age-Standardized Mortality Rates Because of External Causes by Sex and Intent, United States, 1999 to 2020 eFigure 3. Trends in Age-Standardized Mortality Rates Because of External Causes by Age Group, United States, 1999 to 2020 [file jamainternmed-e232509-s001.pdf]

Supplementary Online Content

Lawrence WR, Freedman ND, McGee-Avila JK, et al. Trends in mortality from poisonings, firearms, and all other injuries by intent in the US, 1999-2020. *JAMA Intern Med.* Published July 3, 2023. doi:10.1001/jamainternmed.2023.2509

eTable 1. External Causes of Death ICD-Codes
eTable 2. Leading Causes of Poisoning and All Other Injuries Deaths From 1999-2020
eTable 3. Absolute Differences in External Causes of Death by Sex, Age Group, Race and Ethnicity, and Intent, 1999-2019
eTable 4. Annual Percentage Changes in Death Rates Due to External Causes by Race and Ethnicity, United States, 1999 to 2020
eTable 5. Annual Percentage Changes in Death Rates Due to External Causes by Intent, United States, 1999 to 2020
eTable 6. Annual Percentage Changes in Death Rates Due to External Causes by Sex, United States, 1999 to 2020
eTable 7. Annual Percentage Changes in Death Rates Due to External Causes by Age Group, United States, 1999 to 2020
eTable 8. Annual Percentage Changes in Motor Vehicle-Related Death Rates by Sex, Age Group, and Race and Ethnicity, United States, 1999 to 2020
eTable 9. Annual Percentage Changes in Fall Death Rates by Sex, Age Group, and Race and Ethnicity, United States, 1999 to 2020
eFigure 1. Trends in Age-Standardized Mortality Rates Due to Drug Poisoning by Intent, Unites States, 1999-2020.
eFigure 2. Trends in Age-Standardized Mortality Rates Because of External Causes by Sex and Intent, United States, 1999 to 2020
eFigure 3. Trends in Age-Standardized Mortality Rates Because of External Causes by Age Group, United States, 1999 to 2020

This supplementary material has been provided by the authors to give readers additional information about their work.

© 2023 Lawrence WR et al. *JAMA Internal Medicine.*

eTable 1. External Causes of Death ICD-Codes

Causes/Mechanism	ICD-Codes
Firearm	W32–W34, X72–X74, X93–X95, Y22–Y24, Y35.0
Unintentional	W32–W34
Suicide	X72–X74
Homicide	X93–X95
Undetermined intent	Y22-Y24
Poisoning	X40–X49, X60–X69, X85–X90, Y10–Y19, Y35.2
Unintentional	X40–X49
Suicide	X60–X69
Homicide	X85–X90
Undetermined intent	Y10–Y19
All other Injuries	V01-W31, W35-X39, X50-X59, X70-X71, X76-X84, X91-X92, X96-Y09, Y20-Y21, Y25-Y34, Y35.1, Y35.3-Y36, Y85–Y87, Y89
Unintentional	V01-W31, W35-X39, X50-X59, Y85–Y86
Suicide	X70–X71, X75-X84, Y87.0
Homicide	X91-X92, X96–Y09, Y87.1
Undetermined intent	Y20-Y21, Y25-Y34, Y87.2, Y89.9

Abbreviations: International Statistical Classification of Diseases and Related Health Problems, Tenth Revision, ICD-10

Note: Y35.1 and Y35.3-Y36 are not listed in a specific group as these are deaths due to legal intervention.

© 2023 Lawrence WR et al. *JAMA Internal Medicine.*

eTable 2. Leading Causes of Poisoning and All Other Injuries Deaths from 1999-2020

Causes/Mechanism	n (%)
Poisoning	
Drug overdose	912,670 (91.2%)
All other Injuries	
Motor Vehicle-related	640,425 (29.9%)
Falls	589,641 (27.5%)

© 2023 Lawrence WR et al. *JAMA Internal Medicine.*

eTable 3. Absolute Differences in External Causes of Death by Sex, Age Group, Race and Ethnicity, and Intent, 1999-2019

	Poisoning	Firearm	All other injures
Overall	+31.18	+4.18	+2.57
Sex			
Men	+52.07	+6.78	+12.45
Women	+21.24	+1.05	+1.15
Age Group			
20-39	+36.88	+6.71	-0.64
40-64	+36.33	+3.29	+2.97
≥65	+6.04	+0.64	+8.77
Race and Ethnicity			
Black	+38.85	+15.35	-3.51
White	+37.49	+3.45	5.31
American Indian/ Alaska Native	+65.16	+13.35	2.96
Asian or Pacific Islander	+5.91	-0.81	-3.50
Hispanic/Latino	+17.27	+5.41	-1.62
Intent			
Unintentional	+31.09	-0.14	+0.20
Suicide	-0.29	+1.27	+2.79
Homicide	+0.04	+2.91	-0.59
Undetermined intent	+0.35	+0.01	+0.19

© 2023 Lawrence WR et al. *JAMA Internal Medicine.*

eTable 4. Annual percentage changes in death rates due to external causes by Race and Ethnicity, United States, 1999 to 2020

Cause of death	AAPC from (1999-2020)	APC Segment 1		APC Segment 2		APC Segment 3		APC Segment 4	
Black								NA	NA
Firearm	1.7 (0.7, 2.8)	1999-2014	-0.4 (-1.3, 0.5)	2014-2020	7.2 (4.1, 10.5)	NA	NA	NA	NA
Poison	6.8 (5.4, 8.2)	1999-2013	1.1 (-0.5, 2.7)	2013-2020	19.0 (15.7, 22.3)	NA	NA	NA	NA
All other injuries	-0.4 (-1.9, 1.0)	1999-2006	-1.5 (-2.7, -0.3)	2006-2009	-7.3 (-15.6, 1.7)	2009-2014	-0.0 (-2.9, 3.0)	2014-2020	4.2 (2.6, 5.8)
White								NA	NA
Firearm	1.3 (1.0, 1.6)	1999-2006	0.1 (-0.6, 0.8)	2006-2020	1.9 (1.7, 2.2)	NA	NA	NA	NA
Poison	7.5 (5.9, 9.1)	1999-2004	11.6 (5.0, 18.7)	2004-2020	6.2 (5.5, 7.0)	NA	NA	NA	NA
All other injuries	0.6 (0.1, 1.1)	1999-2006	1.2 (0.8, 1.7)	2006-2009	-4.0 (-7.2, -0.7)	2009-2020	1.5 (1.3, 1.7)	NA	NA
American Indian/ Alaska Native									
Firearm	3.0 (1.6, 4.4)	1999-2014	1.3 (0.0, 2.6)	2014-2020	7.4 (3.2, 11.8)	NA	NA	NA	NA
Poison	9.2 (7.4, 10.9)	1999-2009	12.4 (10.1, 14.7)	2009-2018	3.7 (1.9, 5.6)	2018-2020	18.9 (4.2, 35.8)	NA	NA
All other injuries	0.4 (-1.4, 2.2)	1999-2006	0.6 (-1.1, 2.4)	2006-2009	-5.6 (-16.9, 7.3)	2009-2020	1.9 (1.1, 2.7)	NA	NA
Asian or Pacific Islander									
Firearm	-0.7 (-1.5, 0.0)	1999-2010	-3.2 (-4.3, -2.1)	2010-2020	2.1 (0.9, 3.2)	NA	NA	NA	NA
Poison	6.4 (5.5, 7.3)	1999-2014	4.6 (3.5, 5.6)	2014-2020	11.1 (8.7, 13.6)	NA	NA	NA	NA
All other injuries	-0.6 (-1.6, 0.4)	1999-2005	0.1 (-1.0, 1.4)	2005-2008	-5.0 (-11.5, 2.0)	2008-2020	0.2 (-0.2, 0.6)	NA	NA
Hispanic/Latino									
Firearm	-0.3 (-1.0, 0.4)	1999-2014	-2.4 (-3.1, -1.8)	2014-2020	5.2 (3.1, 7.4)	NA	NA	NA	NA
Poison	5.7 (4.7, 6.7)	1999-2014	2.5 (1.4, 3.6)	2014-2020	14.3 (11.6, 17.1)	NA	NA	NA	NA
All other injuries	-0.4 (-0.9, 0.2)	1999-2006	0.1 (-0.6, 0.9)	2006-2010	-6.6 (-9.0, -4.1)	2010-2020	1.9 (1.5, 2.2)	NA	NA

Segments were chosen by Joinpoint regression.

Abbreviations: AAPC, Average annual percentage change; APC, Annual percentage change; NA, not applicable

Note: a specific segment assumes that the trend is continuous at the joinpoint

© 2023 Lawrence WR et al. *JAMA Internal Medicine.*

eTable 5. Annual percentage changes in death rates due to external causes by intent, United States, 1999 to 2020

Cause of death	AAPC from (1999-2020)	APC Segment 1		APC Segment 2		APC Segment 3	
Firearm	1.1 (0.7, 1.5)	1999-2012	0.1 (-0.3, 0.5)	2012-2020	2.8 (2.0, 3.6)	NA	NA
Unintentional	-3.7 (-4.3, -3.2)	1999-2020	-3.7 (-4.3, -3.2)	NA	NA	NA	NA
Suicide	0.7 (0.3, 1.0)	1999-2006	-0.7 (-1.2, -0.2)	2006-2018	1.7 (1.5, 1.9)	2018-2020	-0.9 (-3.9, 2.3)
Homicide	1.6 (0.5, 2.7)	1999-2014	-0.4 (-1.3, 0.5)	2014-2020	6.9 (3.5, 10.4)	NA	NA
Undetermined intent	0.5 (-0.6, 1.6)	1999-2005	-4.7 (-8.1, -1.1)	2005-2020	2.6 (1.7, 3.5)	NA	NA
Poison	7.0 (5.4, 8.7)	1999-2006	8.9 (5.4, 12.5)	2006-2013	3.2 (-0.1, 6.6)	2013-2020	9.1 (6.9, 11.4)
Unintentional	8.1 (7.4, 8.9)	1999-2020	8.1 (7.4, 8.9)	NA	NA	NA	NA
Suicide	-0.4 (-0.8, 0.0)	1999-2008	2.0 (1.5, 2.5)	2008-2016	-0.5 (-1.2, 0.2)	2016-2020	-5.4 (-7.0, -3.8)
Homicide	—	—	—	NA	NA	NA	NA
Undetermined intent	1.7 (0.2, 3.2)	1999-2003	10.1 (3.2, 17.4)	2003-2013	-2.9 (-4.4, -1.4)	2013-2020	4.0 (1.6, 6.3)
All other injuries	0.2 (-0.3, 0.8)	1999-2006	0.7 (0.2, 1.2)	2006-2009	-5.2 (-8.9, -1.3)	2009-2020	1.5 (1.2, 1.7)
Unintentional	-0.0 (-0.7, 0.6)	1999-2006	0.6 (-0.0, 1.2)	2006-2009	-6.4 (-10.7, -1.9)	2009-2020	1.4 (1.0, 1.7)
Suicide	3.0 (2.5, 3.4)	1999-2018	3.5 (3.3, 3.7)	2018-2020	-1.7 (-6.4, 3.3)	NA	NA
Homicide	-1.5 (-2.2, -0.8)	1999-2018	-2.2 (-2.4, -2.0)	2018-2020	5.0 (-3.1, 13.8)	NA	NA
Undetermined intent	1.8 (0.7, 3.0)	1999-2009	2.8 (1.8, 3.8)	2009-2014	-2.7 (-6.2, 0.9)	2014-2020	4.1 (1.7, 6.6)

Segments were chosen by Joinpoint regression.

Abbreviations: AAPC, Average annual percentage change; APC, Annual percentage change; NA, not applicable

Note: a specific segment assumes that the trend is continuous at the joinpoint

"—" = not calculable

© 2023 Lawrence WR et al. *JAMA Internal Medicine.*

eTable 6. Annual percentage changes in death rates due to external causes by sex, United States, 1999 to 2020

Cause of death	AAPC from (1999-2020)	APC Segment 1		APC Segment 2		APC Segment 3		APC Segment 4	
Men									
Firearm	1.0 (0.6, 1.4)	1999-2013	0.0 (-0.4, 0.4)	2013-2020	3.0 (2.0, 4.0)	NA	NA	NA	NA
Poison	6.6 (5.5, 7.8)	1999-2014	5.0 (3.8, 6.2)	2014-2020	10.8 (7.7, 14.0)	NA	NA	NA	NA
All other injuries	0.2 (-0.4, 0.8)	1999-2006	0.7 (0.2, 1.2)	2006-2009	-4.4 (-7.9, -0.8)	2009-2013	0.2 (-1.6, 2.1)	2013-2020	1.8 (1.3, 2.3)
Women									
Firearm	1.2 (0.8, 1.5)	1999-2008	-0.6 (-1.3, 0.1)	2008-2020	2.5 (2.1, 2.9)	NA	NA	NA	NA
Poison	6.7 (5.5, 7.8)	1999-2005	11.4 (7.2, 15.7)	2005-2020	4.8 (4.2, 5.5)	NA	NA	NA	NA
All other injuries	0.3 (-0.1, 0.7)	1999-2015	0.8 (0.1, 1.5)	2005-2009	-4.3 (-6.3, -2.2)	2009-2020	1.7 (1.5, 2.0)	NA	NA

Segments were chosen by Joinpoint regression.

Abbreviations: AAPC, Average annual percentage change; APC, Annual percentage change; NA, not applicable

Note: a specific segment assumes that the trend is continuous at the joinpoint

© 2023 Lawrence WR et al. *JAMA Internal Medicine.*

eTable 7. Annual percentage changes in death rates due to external causes by age group, United States, 1999 to 2020

Cause of death	AAPC from (1999-2020)	APC Segment 1		APC Segment 2		APC Segment 3		APC Segment 4	
Ages 20-39								NA	NA
Firearm	1.3 (0.6. 1.9)	1999-2013	-0.4 (-1.1, 0.2)	2013-2020	4.7 (2.9, 6.5)	NA	NA	NA	NA
Poison	7.2 (5.8, 8.5)	1999-2013	5.7 (4.1, 7.2)	2013-2020	10.2 (7.2, 13.2)	NA	NA	NA	NA
All other injuries	-0.3 (-1.0, 0.4)	1999-2006	0.4 (-0.5, 1.3)	2006-2010	-5.9 (-9.2, -2.6)	2010-2020	1.6 (1.1, 2.2)	NA	NA
Ages 40-64								NA	NA
Firearm	1.2 (0.9, 1.4)	1999-2007	0.7 (0.1, 1.2)	2007-2020	1.5 (1.3, 1.8)	NA	NA	NA	NA
Poison	6.7 (5.3, 8.1)	1999-2007	9.1 (6.7, 11.5)	2007-2013	1.9 (-1.6, 5.5)	2013-2020	8.1 (6.2, 10.0)	NA	NA
All other injuries	0.4 (-0.1, 0.9)	1999-2006	1.2 (0.8, 1.6)	2006-2009	-3.9 (-6.7, -1.0)	2009-2013	-0.1 (-1.6, 1.4)	2013-2020	1.8 (1.4, 2.2)
Ages ≥65									
Firearm	0.2 (-0.2, 0.6)	1999-2006	-1.4 (-2.1, -0.8)	2006-2018	1.4 (1.1, 1.7)	2018- 2020	-1.5 (-5.1, 2.2)	NA	NA
Poison	4.9 (4.5,5.4)	1999-2016	4.1 (3.7, 4.4)	2016-2020	8.8 (6.6, 11.0)	NA	NA	NA	NA
All other injuries	0.6 (0.0, 1.2)	1999-2005	1.0 (0.4, 1.7)	2005-2008	-4.5 (-8.2, -0.6)	2008-2020	1.7 (1.5, 1.9)	NA	NA

Segments were chosen by Joinpoint regression.
Abbreviations: AAPC, Average annual percentage change; APC, Annual percentage change; NA, not applicable
Note: a specific segment assumes that the trend is continuous at the joinpoint

© 2023 Lawrence WR et al. *JAMA Internal Medicine.*

eTable 8. Annual percentage changes in motor vehicle-related death rates by sex, age group, and race and ethnicity, United States, 1999 to 2020

Cause of death	AAPC from (1999-2020)	APC Segment 1		APC Segment 2		APC Segment 3		APC Segment 4		APC Segment 5	
Motor Vehicle-related deaths	-1.0 (-1.9, -0.1)	1999-2006	0.2 (-1.0, 1.4)	2006-2010	-8.1 (-12.2, -3.8)	2010-2020	1.1 (0.3, 1.8)	NA	NA	NA	NA
Sex											
Men	-0.8 (-1.8, 0.2)	1999-2006	0.6 (-0.7, 2.0)	2006-2010	-8.1 (-12.6, -3.4)	2010-2020	1.2 (0.4, 2.0)	NA	NA	NA	NA
Women	-1.6 (-2.8, -0.3)	1999-2006	-0.9 (-2.0, 0.2)	2006-2009	-9.4 (-16.5, -1.6)	2009-2014	-1.3 (-3.9, 1.3)	2014-2020	1.5 (0.1, 3.0)	NA	NA
Age Group											
20-39	-1.0 (-2.1, 0.2)	1999-2006	1.0 (-0.4, 2.5)	2006-2010	-9.4 (-14.3, -4.1)	2010-2020	1.2 (0.2, 2.1)	NA	NA	NA	NA
40-64	-0.4 (-1.4,0.5)	1999-2006	0.9 (-0.4, 2.1)	2006-2010	-7.6 (-11.8, -3.2)	2010-2020	1.6 (0.9, 2.4)	NA	NA	NA	NA
≥65	-2.3 (-4.1, -0.5)	1999-2004	-1.5 (-3.7, 0.8)	2004-2009	-5.6 (-8.8, -2.3)	2009-2014	-2.3 (-5.8, 1.4)	2014-2017	3.5 (-7.1, 15.3)	2017-2020	-3.9 (-8.8, 1.2)
Race and Ethnicity											
Black	0.2 (-1.2, 1.6)	1999-2006	-0.7 (-2.8, 1.4)	2006-2011	-6.6 (-11.3, -1.7)	2011-2020	5.0 (3.5, 6.4)	NA	NA	NA	NA
White	-1.1 (-2.0, -0.2)	1999-2006	0.6 (-0.4, 1.7)	2006-2010	-7.3 (-11.2, -3.2)	2010-2020	0.3 (-0.5, 1.0)	NA	NA	NA	NA
American Indian/ Alaska Native	-0.8 (-2.7, 1.1)	1999-2003	4.2 (-2.7, 11.7)	2003-2009	-6.1 (-10.7, -1.2)	2009-2020	0.4 (-1.1, 1.9)	NA	NA	NA	NA
Asian or Pacific Islander	-3.1 (-5.3, -0.8)	1999-2006	-2.6 (-4.8, -0.4)	2006-2009	-11.3 (-25.0, 4.9)	2009-2020	-1.0 (-2.1, 0.1)	NA	NA	NA	NA
Hispanic/Latino	-1.3 (-2.6, 0.0)	1999-2006	-0.4 (-2.1, 1.4)	2006-2010	-10.9 (-16.5, -4.9)	2010-2020	2.2 (1.3, 3.1)	NA	NA	NA	NA

Segments were chosen by Joinpoint regression.
Abbreviations: AAPC, Average annual percentage change; APC, Annual percentage change; NA, not applicable
Note: a specific segment assumes that the trend is continuous at the joinpoint

© 2023 Lawrence WR et al. *JAMA Internal Medicine.*

eTable 9. Annual percentage changes in fall death rates by sex, age group, and race and ethnicity, United States, 1999 to 2020

Cause of death	AAPC from (1999-2020)	APC Segment 1		APC Segment 2		APC Segment 3		APC Segment 4		APC Segment 5	
Falls	3.6 (3.4, 3.9)	1999-2007	5.2 (4.6, 5.8)	2007-2020	2.7 (2.4, 2.9)	NA	NA	NA	NA	NA	NA
Sex											
Men	3.0 (2.7, 3.3)	1999-2007	4.3 (3.6, 5.0)	2007-2020	2.2 (2.0, 2.5)	NA	NA	NA	NA	NA	NA
Women	4.2 (3.9, 4.5)	1999-2007	6.3 (5.5, 7.0)	2007-2020	3.0 (2.7, 3.2)	NA	NA	NA	NA	NA	NA
Age Group											
20-39	0.4 (-0.1, 1.0)	1999-2011	-0.8 (-1.5, -0.1)	2011-2020	2.1 (1.1, 3.1)	NA	NA	NA	NA	NA	NA
40-64	2.1 (1.2, 3.0)	1999-2006	3.5 (2.6, 4.4)	2006-2010	-0.0 (-2.8, 2.9)	2010-2014	2.5 (-0.2, 5.3)	2014-2018	-0.4 (-2.9, 2.3)	2018-2020	5.6 (0.6, 10.8)
≥65	4.2 (3.9, 4.4)	1999-2007	6.2 (5.6, 6.9)	2007-2020	3.0 (2.7, 3.2)	NA	NA	NA	NA	NA	NA
Race and Ethnicity											
Black	2.0 (1.7, 2.4)	1999-2020	2.0 (1.7, 2.4)	NA	NA	NA	NA	NA	NA	NA	NA
White	4.0 (3.8,4.3)	1999-2007	5.7 (5.0, 6.4)	2007-2020	3.0 (2.8, 3.3)	NA	NA	NA	NA	NA	NA
American Indian/ Alaska Native	2.5 (1.8, 3.3)	1999-2020	2.5 (1.8, 3.3)	NA	NA	NA	NA	NA	NA	NA	NA
Asian or Pacific Islander	2.2 (1.5, 3.0)	1999-2005	4.3 (1.6, 7.0)	2005-2020	1.4 (1.0, 1.8)	NA	NA	NA			
Hispanic/Latino	2.3 (1.6, 3.0)	1999-2005	4.6 (2.3, 7.0)	2005-2020	1.4 (1.0, 1.8)	NA	NA	NA	NA	NA	NA

Segments were chosen by Joinpoint regression.
Abbreviations: AAPC, Average annual percentage change; APC, Annual percentage change; NA, not applicable
Note: a specific segment assumes that the trend is continuous at the joinpoint

© 2023 Lawrence WR et al. *JAMA Internal Medicine.*

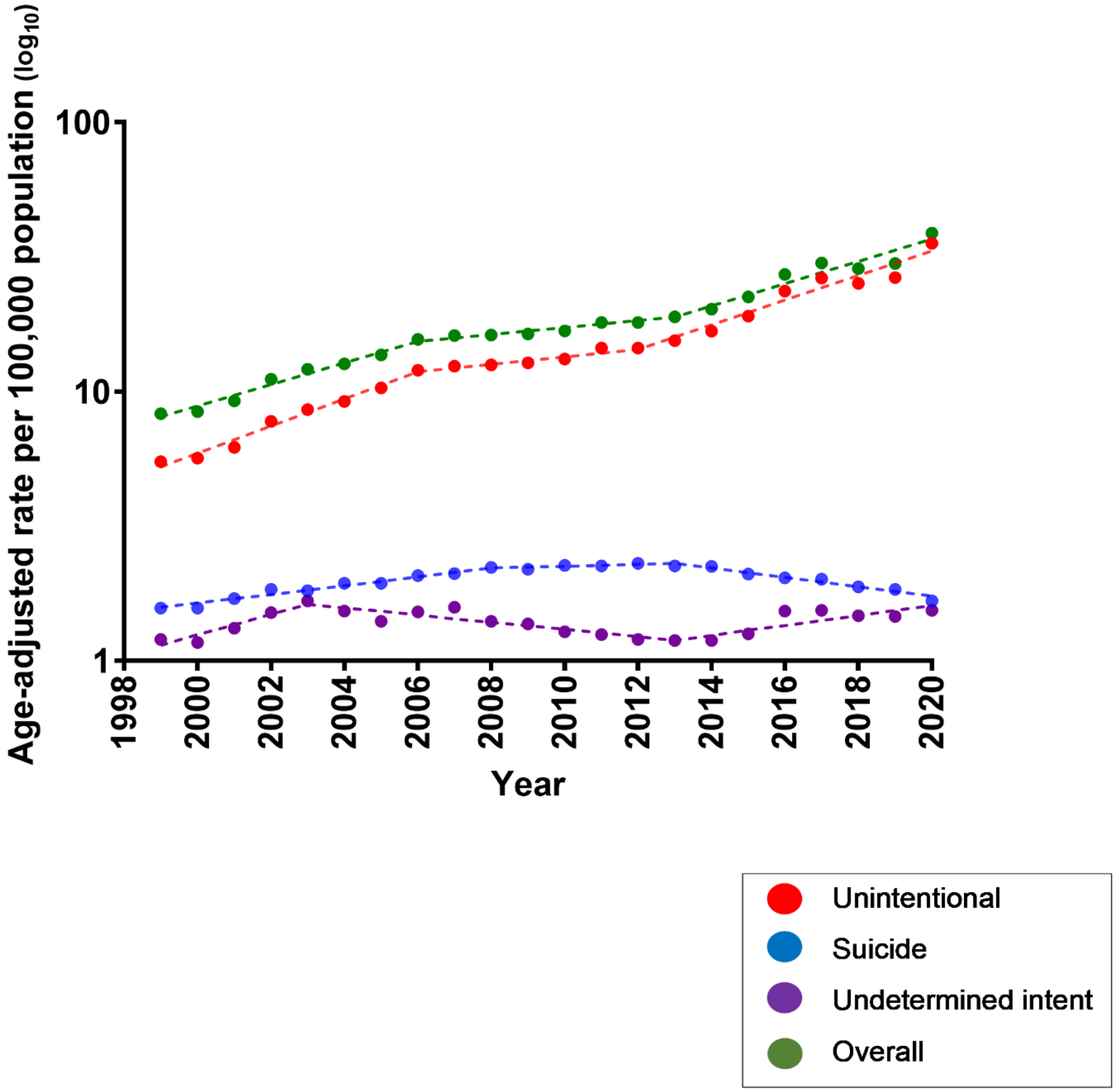

eFigure 1. Trends in Age-Standardized Mortality Rates due to Drug Poisoning by Intent, Unites States, 1999-2020.

Note: "---" represents modeled age-adjusted and "●" represents observed age-adjusted rates.

© 2023 Lawrence WR et al. *JAMA Internal Medicine.*

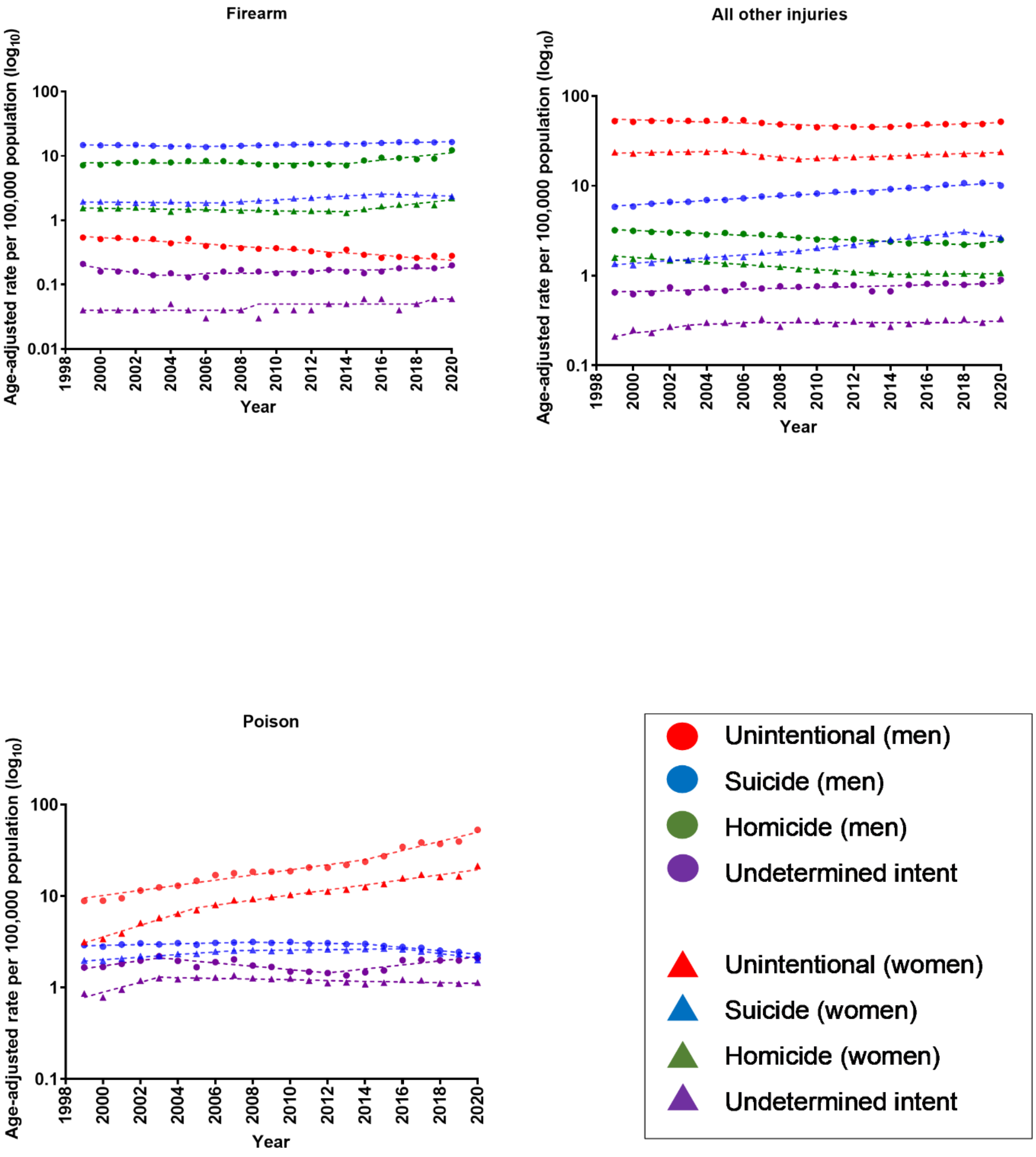

eFigure 2. Trends in Age-Standardized Mortality Rates Because of External Causes by Sex and Intent, United States, 1999 to 2020

Note: "---" represents modeled age-adjusted and "●" represents observed age-adjusted rates.

© 2023 Lawrence WR et al. *JAMA Internal Medicine.*

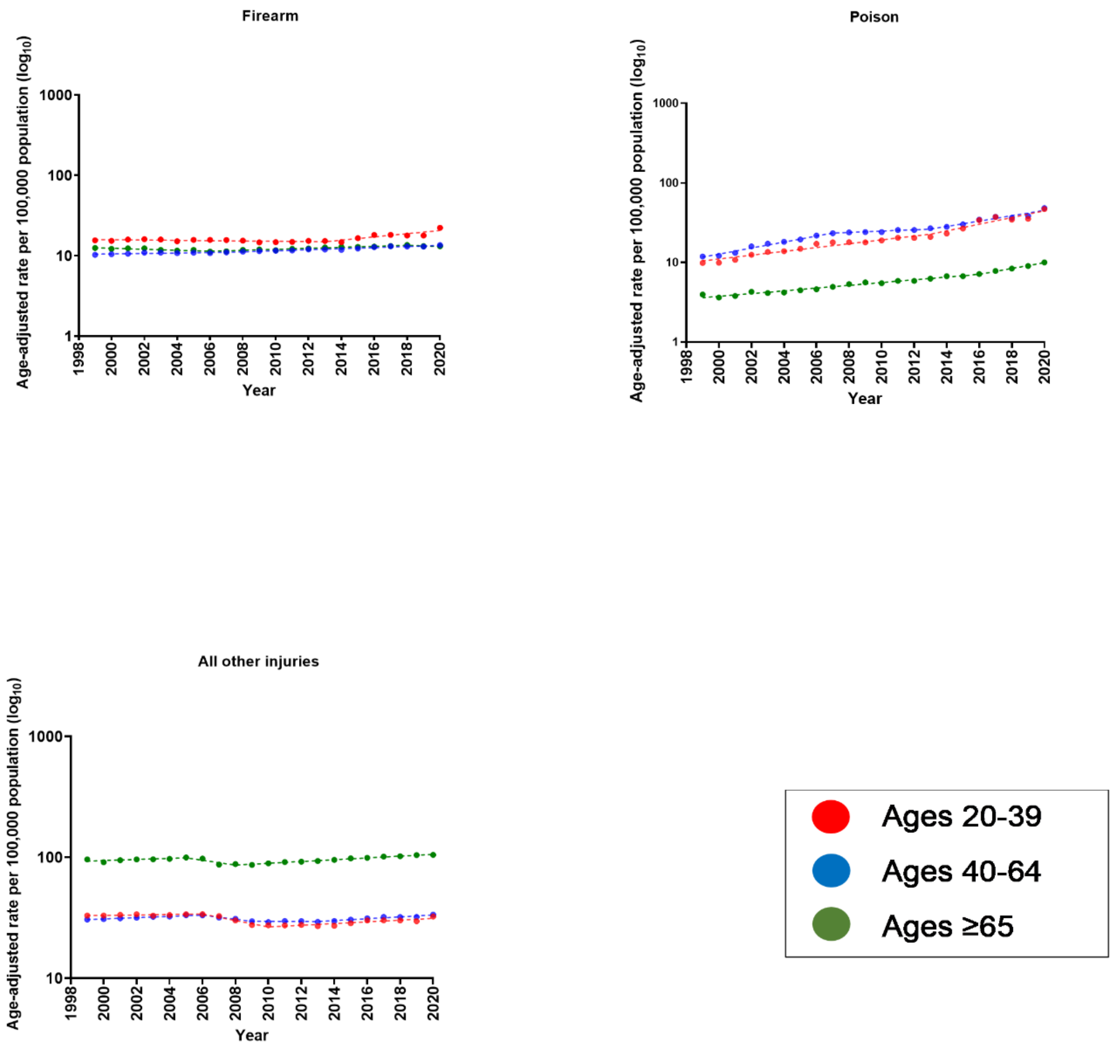

eFigure 3. Trends in Age-Standardized Mortality Rates Because of External Causes by Age Group, United States, 1999 to 2020

Note: "---" represents modeled age-adjusted and "●" represents observed age-adjusted rates.

© 2023 Lawrence WR et al. *JAMA Internal Medicine.*
