# Supplementary material for: Trends in Mortality From Poisonings, Firearms, and All Other Injuries by Intent in the US, 1999-2020
Source: JAMA Intern Med. 2023 Jul 3;183(8):849–56. doi: 10.1001/jamainternmed.2023.2509 (PMC10318548; doi:10.1001/jamainternmed.2023.2509)
Supplement: Supplement 2. — Data Sharing Statement [file jamainternmed-e232509-s002.pdf]

Data Sharing Statement

Lawrence. Trends in Mortality From Poisonings, Firearms, and All Other Injuries by Intent in the US, 1999-2020. *JAMA Intern Med*. Published July 03, 2023.
doi:10.1001/jamainternmed.2023.2509

Data
Data available: Yes
Data types: Deidentified participant data
How to access data: CDC Wide-ranging ONline Data for Epidemiologic Research (WONDER) https://wonder.cdc.gov/ucd-icd10-expanded.html
When available: With publication

Supporting Documents
Document types: None

Additional Information
Who can access the data: Anyone through CDC Wide-ranging ONline Data for Epidemiologic Research (WONDER) https://wonder.cdc.gov/ucd-icd10-expanded.html
Types of analyses: CDC Wide-ranging ONline Data for Epidemiologic Research (WONDER) available for any purpose
Mechanisms of data availability: Data user must electronically agree to abide by the terms of data use stated. See below snippet "By clicking the "I Agree" button I signify that I will abide by the terms of data use stated above and understand the sanctions and legal penalties for violation of these terms of use."
